# Diagnostic value of serum leptin and a promising novel diagnostic model for sepsis

**DOI:** 10.3892/etm.2014.1506

**Published:** 2014-01-27

**Authors:** MINGYI CHEN, BIN WANG, YAPING XU, ZIHUI DENG, HUI XUE, LUHUAN WANG, LEI HE

**Affiliations:** 1 Department of Hepatobiliary Surgery, Chinese People’s Liberation Army General Hospital, Beijing 100853, P.R. China; 2 Department of Critical Care Medicine, Chinese People’s Liberation Army General Hospital, Beijing 100853, P.R. China; 3 Center of Inspection of Clinical Division, Chinese People’s Liberation Army General Hospital, Beijing 100853, P.R. China; 4 Research Laboratory of Biochemistry, Basic Medical Institute, Chinese People’s Liberation Army General Hospital, Beijing 100853, P.R. China

**Keywords:** sepsis, leptin, systemic inflammatory response syndrome, diagnosis

## Abstract

Diagnosis of sepsis in critically ill patients is important to reduce morbidity and mortality. The present study was conducted to determine the role of serum leptin in the early diagnosis of sepsis and to establish a diagnostic model for sepsis. A retrospective study was conducted of 331 patients from an intensive care unit. All patients underwent consistent blood collection at 6:00 a.m. every morning after fasting. Serum leptin concentrations and additional markers of sepsis were compared between the sepsis group (n=128) and the non-sepsis group (n=203). Septic patients displayed significantly higher leptin serum concentrations compared with those of the non-sepsis group (mean concentration, 11.67 versus 4.824 mg/dl; P<0.001). The leptin levels in male patients were higher than those in female patients, particularly in the sepsis group. The accuracy of serum leptin levels in distinguishing septic patients from non-septic patients was 76%, and the area under the receiver operating characteristic (ROC) curve of serum leptin was ≤0.8. Additional markers of inflammation in the sepsis group were also significantly higher than those in the non-sepsis group. Positive correlations were identified between leptin and body temperature, heart rate and creatinine levels. Therefore, a prognostic model comprising a combination of leptin with temperature, platelet count, white blood cell count and heart rate was evaluated as an effective logistic regression model for the diagnosis of sepsis. The logistic regression output cut-off value was 0.46 and the area under the ROC curve was 0.953 (P<0.0001). It may be concluded that leptin is a valuable marker in the diagnosis of sepsis and the proposed prognostic model is an effective logistic regression model for the diagnosis of sepsis. The prognostic model is able to aid the differentiation of septic patients from non-septic patients.

## Introduction

Sepsis is a systemic inflammatory reaction that is triggered by an infective agent (such as bacteria, viruses, fungi or parasites) (1). Severe sepsis may result in systemic inflammation, multi-organ failure and septic shock (2). It is one of the major health concerns worldwide and also the predominant reason for intensive care unit (ICU) admission (3). There are 750,000 cases of severe sepsis diagnosed every year in the United States, accompanied by unacceptably high mortality rates (4,5). Sepsis-induced mortality rates are as high as 30–50% in developed countries. With the rapidly increasing incidence, high mortality rates, complex pathophysiology and overall difficulties in its treatment, sepsis is becoming an important focus for researchers and clinicians (6–9).

Infections and sepsis are accompanied by clinical signs such as leukocytosis, changes in body temperature and the development of tachycardia. However, these classic indicators of systemic inflammation are neither sensitive nor specific for sepsis (10). They have only moderate sensitivity and specificity and are not early markers due to the time taken to produce a reaction. Therefore, early markers are useful for the diagnosis and treatment of sepsis and are crucial for overcoming sepsis-associated mortality.

Cytokine levels are an obvious choice as a marker of sepsis. The systemic release of inflammatory cytokines occurs several hours prior to other markers of systemic inflammation, such as acute phase protein release and leucocytosis, suggesting their potential importance as diagnostic parameters in systemic inflammatory response syndrome (SIRS) and sepsis (11,12). When sepsis occurs, multiple redundant inflammatory cytokines are released into the blood stream, including tumor necrosis factor-α (TNF-α), interleukin-6 (IL-6), leptin, C-reactive protein (CRP) and procalcitonin (PCT) (13,14), which are important for mediating the inflammatory response.

The hormone leptin (molecular weight of 16-kDa) is mainly generated by adipocytes and contributes to the regulation of energy balance by informing the brain of the volume of adipose tissue in the body, thereby regulating food intake and energy expenditure (15–17). In addition, previous studies have indicated that leptin may be classified as a cytokine, and is involved in cell-mediated immunity and cytokine crosstalk (18–22). The present study was conducted to determine the role of serum leptin in the early diagnosis of sepsis and to explore its correlation with additional biomarkers of sepsis and/or multiple organ failure. This knowledge is likely to be helpful in understanding the precise mechanisms of sepsis and also potentially contributes to new diagnostic approaches.

A large-sample-sized study of septic patients from a medical ICU was conducted. Serum leptin concentrations were measured during treatment in the ICU to investigate whether leptin is activated in critical illness, has diagnostic values for sepsis and/or multiple organ failure and whether leptin may serve as a prognostic predictor for survival in the ICU and long term.

## Materials and methods

### Patients

A retrospective study was conducted and a total of 331 patients (115 males and 216 females) were included in the study. As shown in Table I, the median age of the patients was 56 years (range, 6–91 years). The study included 128 patients with sepsis (47 males and 81 females) with a median age of 53 years. All patients underwent consistent blood collection at 6:00 a.m. every morning after fasting. This study was conducted over a period of 24 months from April 2008 to July 2011 in the ICU of the General Hospital of Chinese People’s Liberation Army (Beijing, China). The study was in accordance with the Helsinki Declaration and approval was obtained from the Ethics Committee of the Chinese PLA General Hospital (project no. 11KMZ04). The subjects and their families were well informed of the details and written informed consent was obtained prior to the study.

### Diagnostic criteria for sepsis

The gold standard for the diagnosis of sepsis is a positive blood culture. The blood cultures were performed independently more than twice and the same type of positive bacteremia was present at the end of testing. Thus, marked signs of infection had appeared and blood cultures were positive in the enrolled patients of the sepsis group. Previously described criteria (23) for sepsis were considered to be met and at least two of the following four clinical symptoms of SIRS were present: temperature >38°C or <36°C; heart rate >90 beats/min; respiratory rate >20 breaths/min or PaCO_2_ <32 mmHg; and white blood cell (WBC) count >12,000 or <4,000 cells/mm^3^, or >10% immature forms. The patients in the control (non-sepsis) group showed no signs of infection and less than two of the four clinical SIRS symptoms were present.

### Blood sampling

Blood samples were collected and processed within 2 h. Blood was centrifuged at 1,600 × g for 15 min.

### Leptin and CRP determination

Serum leptin and CRP concentrations were determined by quantitative sandwich enzyme immunoassays (Ray Biotech., Inc., Minneapolis, MN, USA) according to the manufacturer’s instructions. The intensity of the color was measured at 450 nm.

### PCT determination

PCT levels were measured using an radioimmunoassay kit (RIN 6025) that utilized specific polyclonal antibodies and human ^125^I-N-procalcitonin with a detection limit of 10 pg/tube (Peninsula Lab., Inc., San Carlos, CA, USA).

### Statistical analysis

Basic statistical analyses were performed using SPSS software, version 18.0 (SPSS, Inc., Chicago, IL, USA). Categorical data are expressed as total numbers and/or relative frequencies. Continuous data are expressed as the mean ± standard deviation (or mean ± standard error of the mean). The binary logistic regression models were chosen to predict the probability of sepsis. Receiver operating characteristic (ROC) curves were used to evaluate the corresponding balance between sensitivity and specificity over a range of predictive factors (such as leptin only and leptin combined with temperature, platelet count, WBC count and heart rate), and the optimum cut-off for sensitivity and specificity corresponding to maximum area under the ROC (AUC) was determined by selection of the point at which sensitivity plus specificity was maximal.

## Results

### Patient characteristics

A total of 331 patients in the ICU of the General Hospital of Chinese People’s Liberation Army from April 2008 to July 2011, with a median age of 56 years, were studied. The sepsis group consisted of 128 patients from the ICU (47 males and 81 females) with a median age of 53 years (Table I).

### Serum leptin concentrations are elevated in septic patients

**Figure 1:**
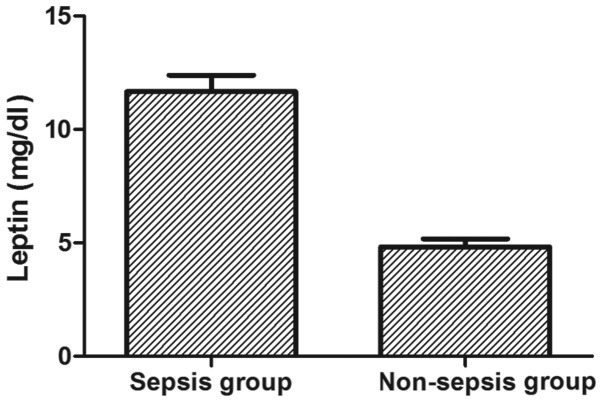
Serum leptin concentrations in the sepsis and non-sepsis groups. Serum leptin concentrations at admission to the medical ICU were significantly (P<0.001) elevated in the sepsis group (n=128) compared with those in the non-sepsis group (n=203). ICU, intensive care unit.

**Figure 2:**
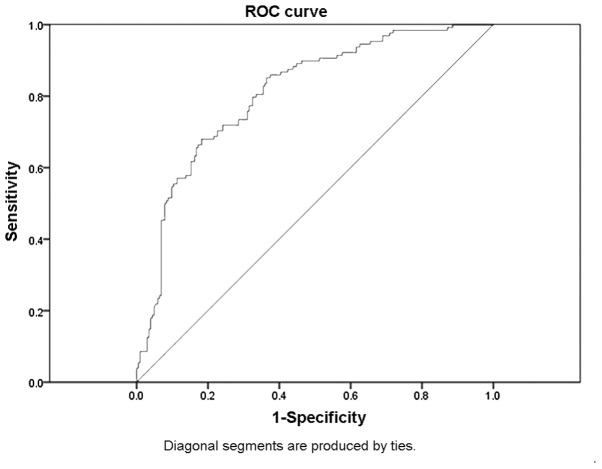
ROC curve for serum leptin in the sepsis group. The reference line represents the ROC curve for a statistical test with no discriminatory ability. The AUC was 0.8 (P<0.0001) and the cut-off level was 5.1 mg/dl (sensitivity, 72% and specificity, 72%) in the sepsis group. ROC, receiver operating characteristic; AUC, area under the ROC.

It was first tested whether the serum concentrations of leptin were elevated in septic patients compared with those in non-septic controls in the ICU. Septic patients displayed significantly higher leptin serum concentrations compared with those of the non-septic controls (mean leptin concentration, 11.67 mg/dl in the sepsis group versus 4.82 mg/dl in the control group; P<0.001; Fig. 1). The data indicate that leptin is associated with sepsis and a subsequent ROC curve analysis revealed that leptin, as an independent indicator, may differentiate septic patients from controls. A cut-off point set at 5.1 mg/dl leptin showed a sensitivity of 72% and a specificity of 72%. The accuracy of serum leptin in distinguishing septic patients from non-septic patients was 76%, and the AUC of serum leptin was ≤0.8 (Fig. 2).

### Serum leptin levels in males are higher than those in females

**Figure 3:**
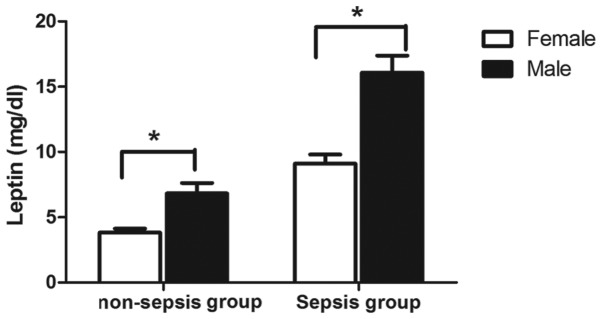
Comparison of serum leptin concentrations between male and female patients in the sepsis and non-sepsis groups. ^*^P<0.001.

The differences in leptin concentrations between male and female patients were evaluated. As shown in Fig. 3, the leptin levels in males were higher than those in females, regardless of the group. The elevation of leptin levels was particularly marked in the sepsis group, in which the mean serum leptin concentrations at admission were 16.07 μg/l in males and 9.1 μg/l in females (P=1.4E-6) (Table II).

### Comparison of clinical indicators between the sepsis and non-sepsis groups

Infections and sepsis are accompanied by clinical and laboratory signs, such as changes in body temperature, leukocytosis and tachycardia. In the present study, clinical indicators other than leptin were evaluated. As shown in Table III, the leptin concentrations in patients with sepsis were significantly higher than those of the patients in the control group (11.67±0.72 versus 4.82±0.36 mg/dl) (P<0.001). The mean values of the CRP and PCT concentrations and the WBC count in the sepsis group were 9.99 mg/dl, 17.86 ng/ml and 15,620 cells/ml, respectively, versus 3.31 mg/dl, 0.34 ng/ml and 8,360 cells/ml, respectively in the non-sepsis group (P<0.001). Several markers of organ function, such as creatinine levels, the international normalized ratio (INR) and activated partial thromboplastin time (aPTT) were also measured. The mean values of the creatinine concentration and INR in the sepsis group were 99.3 μmol/l and 1.36 respectively, and were significantly higher than those of the controls (P<0.001; Table III).

### Correlation between leptin and other indicators of sepsis

The correlations between leptin and additional indicators of sepsis were evaluated. The data revealed no significant correlations between leptin and other inflammatory markers, including CRP, PCT and WBCs. The levels of serum leptin were negatively correlated with platelets and TB (Pearson correlation coefficient = −0.119 and −0.138; P=0.03 and 0.016, respectively), and positively correlated with body temperature, heart rate and creatinine levels (Pearson correlation coefficient = 0.303, 0.111 and 0.286; P=1.9E-8, 0.044 and 4.6E-7, respectively) (data not shown).

### Leptin serum concentrations indicate sepsis in ICU patients and may be used in a new diagnostic model for sepsis

**Figure 4:**
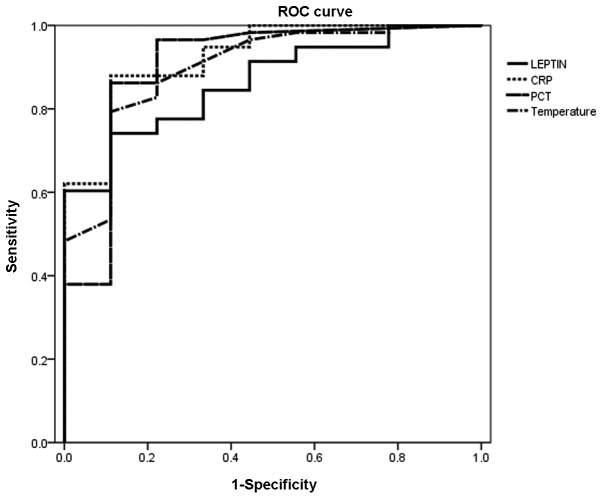
ROC curve analyses comparing the diagnostic power of leptin for predicting sepsis in patients in a medical ICU (AUC=0.864) with classic markers of inflammation and infection, such as CRP (AUC=0.925), PCT (AUC=0.904) and temperature (AUC=0.898). ROC, receiver operating characteristic; AUC, area under the ROC; CRP, C-reactive protein; PCT, procalcitonin.

Based on the finding that leptin serum concentrations were higher in the septic group than in the control group, the ability of serum leptin levels to identify patients with sepsis in the medical ICU setting was investigated. The diagnostic accuracy of leptin was compared with classic, routinely used markers of inflammation and bacterial infection using ROC curve analyses (24). For leptin, CRP and PCT levels and body temperature, the AUC statistics were 0.864, 0.925, 0.904 and 0.898, respectively (Fig. 4). These data demonstrate that leptin is an effective diagnostic marker for sepsis when compared with classical biomarkers.

**Figure 5:**
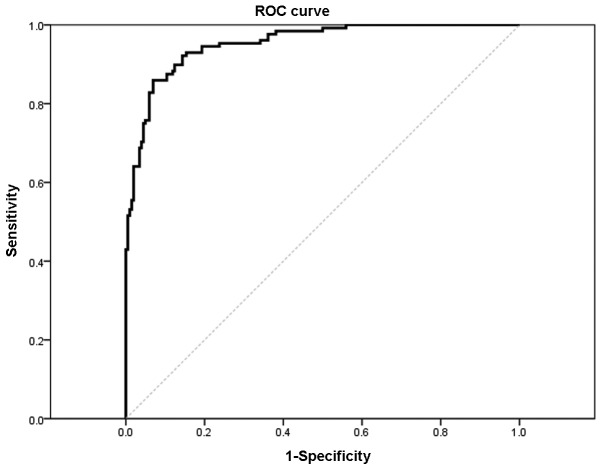
ROC curve in the sepsis group. The reference line represents the ROC curve for a statistical test with no discriminatory ability. The AUC was 0.953 (P<0.0001) and the cut-off of logistic regression output (the odds ratio) equaled 0.46, (sensitivity, 86% and specificity, 93%) in the sepsis group. ROC, receiver operating characteristic; AUC, area under the ROC.

Based on the associations between leptin and markers of organ dysfunction, it was clear that leptin has the potential to facilitate prognosis and guide the treatment of sepsis. Binary logistic regression with a likelihood ratio-based forward stepwise strategy was used to predict the diagnosis of sepsis. The results revealed that leptin, WBCs, platelets, temperature and heart rate were all independent predictors in septic patients with odds ratios of 1.192 [95% confidence interval (CI), 1.119–1.269, P=4E-8), 1.407 (95% CI, 1.251–1.583, P=1.4E-8), 0.992 (95% CI, 0.987–0.996, P=3.9E-4), 3.187 (95% CI, 1.655–6.139, P=0.001) and 1.063 (95% CI, 1.036–1.092, P=4.7E-6), respectively (Table IV). The logistic regression output cut-off value (the odds ratio) equaled 0.46, the model was found to have a sensitivity of 86% and specificity of 93% for sepsis, and the AUC was 0.953 (P<0.0001). The results revealed that leptin, combined with temperature, platelet and WBC counts and heart rate are an effective logistic regression model for the diagnosis of sepsis (Fig. 5).

## Discussion

Early diagnosis of sepsis may be challenging as clinical presentations are often nonspecific, bacterial cultures are time-consuming and laboratory tests lack sensitivity and specificity. In order to reduce the morbidity and mortality associated with sepsis, there is an urgent requirement for effective markers for the diagnosis and monitoring of sepsis. In the present study, it was observed that the serum leptin concentrations were significantly higher in the sepsis group than in the non-sepsis group. Similar results were identified by Arnalich *et al* (25), who also hypothesized that the high leptin levels in survivors with sepsis may represent host defense mechanisms against bacterial infection. Notably, the results of the present study indicated that there were significant differences in leptin concentrations between males and females. The mean serum leptin concentrations in males were 2-fold higher than those of females.

Severe sepsis is defined as sepsis associated with organ dysfunction, hypoperfusion or hypotension, such as serum creatinine levels of >176.8 μmol/l (2.0 mg/dl), an INR of >1.5 or an aPTT of >60 sec (26). In the present study, the mean values of serum creatinine, INR and aPTT were 99.3 μmol/l, 1.36 and 42.9 sec, respectively. Therefore, the patients in the present study did not have severe sepsis. Furthermore, this was supported by the positive correlation that was identified between leptin and creatinine levels, which is an indicator associated with renal function.

The monitoring of serum leptin levels in septic patients is important for the early diagnosis of sepsis and the differentiation between sepsis and SIRS. The results of the present study showed that the AUC statistics for leptin, CRP and PCT levels and temperature were 0.864, 0.925, 0.904 and 0.898, respectively. Thus, leptin may be utilized as a potential diagnostic marker of sepsis with a similar efficacy to other markers, such as CRP and PCT (27–30). It is known that PCT and CRP are useful in the diagnosis of bacterial infection and sepsis, but with limitations. PCT levels are elevated in septic patients and correlated with the severity of sepsis; they usually serve to discriminate between severe sepsis and less severe forms of infection, but do not correlate with early prognosis (31). Concentrations of CRP have been monitored in septic patients, but these concentrations fail to allow immediate diagnosis and prognosis due to the time taken for the body to produce a reaction and the duration of the increased serum concentration (32,33). However, leptin is involved in the network of inflammatory mediators and during SIRS its plasma concentration is increased by the action of these inflammatory mediators (34). Moreover, a significant correlation between leptin and TNF-α has been identified, which indicates that leptin is a crucial regulator in the early inflammatory period (35–37). The exact mechanisms of leptin in sepsis require further study.

As each biomarker has limited sensitivity and specificity, it is likely that a combined panel of novel biomarkers, with or without traditional markers of sepsis is required. Studies using panels of sepsis biomarkers have also provided encouraging results. The present study demonstrated that leptin, in combination with other independent factors, such as temperature, platelet and WBCs and heart rate, may be an effective diagnostic model of sepsis. The logistic regression output cut-off value equaled 0.46 and the model was found to have sensitivity of 86% and specificity of 93%.

A useful sepsis marker is required to not only facilitate the identification of sepsis, but may also be used to guide therapy. The possible role of leptin in the prognosis of sepsis requires further study.

In conclusion, leptin is a valuable marker for the diagnosis of sepsis. A prognostic model has been created that is an effective logistic regression model for the diagnosis of sepsis.

## Figures and Tables

**Table I:** Patient characteristics.

			Age (years)		
			
Group	No.	M/F	Mean ± SD	Range	Median
Sepsis	128	47/81	54±20	10–86	53
Non-sepsis	203	68/135	56±18	6–91	58

Age data are expressed as the mean ± SD, the median and range. SD, standard deviation; M, male; F, female

**Table II:** Serum leptin concentrations in male and female patients with and without sepsis.

Gender	Group	Leptin (μg/l)
Female	Non-sepsis	3.8±0.3
	Sepsis	9.1±0.7
Male	Non-sepsis	6.8±0.8
	Sepsis	16.1±1.3

**Table III:** Comparison of clinical indicators in the sepsis and non-sepsis groups.

Parameters	Sepsis group (Mean ± SD)	Non-sepsis group (Mean ± SD)	P-value
Basic information			
No.	128	203	
Age (years)	54±20	56±18	
Male (n)	47	68	
Female (n)	81	135	
Systemic conditions			
Body temperature (°C)	37.8±0.1	36.9±0.0	<0.001
Heart rate (beats/min)	109±2	87±1	<0.001
Markers of inflammation			
Leptin (mg/dl)	11.67±0.72	4.824±0.355	<0.001
CRP (mg/dl)	9.99±0.69	3.31±1.14	<0.001
PCT (ng/ml)	17.86±5.14	0.34±0.28	<0.001
WBCs (×1000 cells/ml)	15.62±1.17	8.36±0.21	<0.001
Neutrophils (/%)	0.853±0.008	0.792±0.009	<0.001
Markers of organ function			
Creatinine (μmol/l)	99.3±7.6	64.7±2.6	<0.001
INR	1.36±0.06	1.18±0.02	<0.001
TB (μmol/l)	42.7±6.5	46.1±6.7	0.735
aPTT (sec)	42.9±1.3	40.5±1.2	0.192
Platelets (×10^3^/ml)	169±10	198±7	0.011

CRP, C-reactive protein; PCT, procalcitonin; WBCs, white blood cells; INR, internationalized ratio; TB, total bilirubin; aPPT, activated partial thromboplastin time. SD, standard deviation.

**Table IV:** Associations between leptin and other markers in the sepsis group (the output of the logistic regression model).

	Odds ratio	95% CI	P value
Leptin	1.192	1.119–1.269	0.000 (4E-8)
WBCs	1.407	1.251–1.583	0.000 (1.4E-8)
Platelets	0.992	0.987–0.996	0.000 (3.9E-4)
Temperature	3.187	1.655–6.139	0.001
Heart rate	1.063	1.036–1.092	0.000 (4.7E-6)

CI, confidence interval.
